# Arterial spin labelling perfusion during rTMS/iTBS for treatment-resistant depression; a narrative review

**DOI:** 10.1192/j.eurpsy.2026.11398

**Published:** 2026-07-31

**Authors:** N. Skandali

**Affiliations:** Centre for Neuroimaging Sciences Institute of Psychiatry, Psychology & Neuroscience, King’s College London, London, United Kingdom

## Abstract

**Introduction:**

Repetitive transcranial magnetic stimulation (rTMS) and its accelerated protocol, intermittent theta-burst stimulation (iTBS), show established efficacy above placebo for treatment-resistant depression (TRD) (Saelens et al. NPP 2025;50(6):913–919), yet response rates remain suboptimal and biomarkers of treatment response are lacking. Arterial spin labelling (ASL) is a non-invasive MRI technique that provides quantitative, repeatable measures of cerebral blood flow (CBF); it is quick, low-cost and has high temporal and spatial resolution (Ho, J Neuroradiol 2018;45(5):276–289), making it well suited for tracking neuroplastic and circuit-level changes induced by multiple sessions of brain stimulation. ASL studies in TRD show cortico-striatal-thalamic abnormalities and sgACC hyperperfusion in depression (Duhameau et al. Psychiatry Res Neuroimaging 2010;182(2):111–116). The effects of rTMS/iTBS on brain perfusion using ASL have been assessed across small, methodologically heterogeneous studies, limiting conclusions about robust neural signatures of response.

**Objectives:**

To narratively synthesise ASL-measured perfusion changes during or after rTMS/iTBS specifically in TRD, highlighting convergent findings, inconsistencies, and methodological priorities for future research.

**Methods:**

Narrative review of peer-reviewed human studies in adults with TRD (or MDD cohorts reporting TRD sub-analyses) that included ASL-derived CBF measures in the context of rTMS/iTBS/accelerated protocols. Databases searched: MEDLINE, EMBASE, PsycINFO, Scopus and Web of Science (inception to present), supplemented by reference-list checks. Case reports and non-English abstracts were excluded. Extracted data included stimulation parameters, ASL method (e.g., pCASL), scanner field strength, scan timing (acute vs post-course), regions of interest (ROIs), direction of effect, and clinical outcome.

**Results:**

Approximately seven studies met inclusion criteria, most using pCASL at 3 T with post-course scanning. ROIs frequently included left dorsolateral prefrontal cortex (DLPFC) and subgenual anterior cingulate cortex (sgACC), with some network-level analyses (salience and default mode networks). Consistently reported findings include increased DLPFC CBF following excitatory prefrontal stimulation and reduced sgACC perfusion in clinical responders, though results are inconsistent. Heterogeneity in stimulation protocols, scan timing, ASL sequences, and ROI definitions limits comparability and precludes meta-analysis.

**Conclusions:**

ASL is a promising tool for detecting rTMS/iTBS-related perfusion modulation in TRD and may hold potential as biomarker for treatment response. Future studies should standardise stimulation targets/parameters, pre-specify scan timing, report ASL acquisition/processing transparently (including tissue correction), and harmonise ROI definitions to enable pooling and effect-size estimation.

**Disclosure of Interest:**

None Declared

